# A High-Throughput HIV-1 Drug Screening Platform, Based on Lentiviral Vectors and Compatible with Biosafety Level-1

**DOI:** 10.3390/v12050580

**Published:** 2020-05-25

**Authors:** Bernhard Ellinger, Daniel Pohlmann, Jannis Woens, Felix M. Jäkel, Jeanette Reinshagen, Carol Stocking, Vladimir S. Prassolov, Boris Fehse, Kristoffer Riecken

**Affiliations:** 1 Department ScreeningPort, Fraunhofer Institute for Molecular Biology and Applied Ecology IME, 22525 Hamburg, Germany; bernhard.ellinger@ime.fraunhofer.de (B.E.); jeanette.reinshagen@ime.fraunhofer.de (J.R.); 2 Fraunhofer Cluster of Excellence Immune-Mediated Diseases CIMD, Partner site Hamburg, 22525 Hamburg, Germany; 3 Research Department Cell and Gene Therapy, Department of Stem Cell Transplantation, University Medical Center Hamburg-Eppendorf (UKE), 20246 Hamburg, Germany; daniel.pohlmann@outlook.com (D.P.); j.woens@uke.de (J.W.); fmjaekel@gmail.com (F.M.J.); c.stocking@uke.de (C.S.); 4 Heinrich-Pette-Institute, Leibniz Institute for Experimental Virology, 20251 Hamburg, Germany; 5 Engelhardt-Institute of Molecular Biology, Russian Academy of Sciences, 117984 Moscow, Russia; prassolov45@mail.ru; 6 German Center for Infection Research (DZIF), Partner site Hamburg, 20246 Hamburg, Germany

**Keywords:** HIV-1 drug development, BSL-1 screening platform, high-throughput screening, lentiviral vectors, mCat1 expressing PM1 T cell line, LeGO vectors

## Abstract

HIV-1 infection is a complex, multi-step process involving not only viral, but also multiple cellular factors. To date, drug discovery methods have primarily focused on the inhibition of single viral proteins. We present an efficient and unbiased approach, compatible with biosafety level 1 (BSL-1) conditions, to identify inhibitors of HIV-1 reverse transcription, intracellular trafficking, nuclear entry and genome integration. Starting with a fluorescent assay setup, we systematically improved the screening methodology in terms of stability, efficiency and pharmacological relevance. Stability and throughput were optimized by switching to a luciferase-based readout. BSL-1 compliance was achieved without sacrificing pharmacological relevance by using lentiviral particles pseudo-typed with the mouse ecotropic envelope protein to transduce human PM1 T cells gene-modified to express the corresponding murine receptor. The cellular assay was used to screen 26,048 compounds selected for maximum diversity from a 200,640-compound in-house library. This yielded z’ values greater than 0.8 with a hit rate of 3.3% and a confirmation rate of 50%. We selected 93 hits and enriched the collection with 279 similar compounds from the in-house library to identify promising structural features. The most active compounds were validated using orthogonal assay formats. The similarity of the compound profiles across the different platforms demonstrated that the reported lentiviral assay system is a robust and versatile tool for the identification of novel HIV-1 inhibitors.

## 1. Introduction

The pharmacological battle against HIV-1 began 30 years ago with the application of azidothymidine (AZT), a nucleoside analog reverse-transcriptase inhibitor (NRTI), whose antiretroviral activity had already been described in the 1970s [1]. The identification of reverse transcriptase as the target for this class of molecules led researchers to focus on enzyme-based assay systems, which resulted in the development of non-nucleotide reverse-transcriptase inhibitors (NNRTIs) [2]. Similar enzyme-based screens were used to identify HIV-1 protease inhibitors, but enzyme-based assays in general have intrinsic difficulties [3]. HIV-1 has a 100,000-fold higher mutation rate per base and replication cycle than yeast, thereby generating frequent mutants that escape small-molecule inhibitors, which rely on only a few interactions to bind to their target protein [4,5]. The limited number of viral proteins also makes the identification of novel target sites for screening extremely challenging. A truly unbiased cellular screening that is not limited to certain target classes would provide an alternative, but screening against HIV-1 still relies mostly on target-centered approaches.

The limitations described above have restricted the choice of target classes for HIV therapy, and thus current anti-retroviral therapy (ART) relies on combinations of drugs against three viral proteins and very few entry inhibitors. The availability of ART has greatly reduced mortality and morbidity of HIV infection. However, on a global scale, infection rates are decreasing much slower than anticipated and are even rising in Eastern Europe and central Asia [6]. Additionally, pretreatment NNRTI resistance is increasing worldwide, calling for the development of novel, small-molecule treatment options, as only this drug type will have the chance of being cost-effective and readily distributable in remote areas. With currently 36.7 million people living with HIV and one million deaths annually, adequate HIV treatment is by far not available to every infected person today [7]. The access to ART is crucial to control transmission and to reach the 90-90-90 goal of UNAIDS, but only 37% of infected adults and 24% of the children receive it [8]. The disease is therefore far from being under control, and multiple efforts are required to achieve effective containment.

A promising approach to target HIV is the development of new small-molecule drugs against targets associated with a low likelihood of resistance development. However, as mentioned above, the limited number of proteins encoded by HIV, coupled to their extensive application in previous screening programs and their high mutation rate, make them less attractive for novel drug discovery approaches. An alternative strategy would be the development of modulators against the network of host proteins necessary for HIV infection and pathogenicity. The September 2017 release of the HIV-1 Interaction Database listed 8005 interactions involving a total of 3859 cellular proteins, including 1595 interactions necessary for virus replication [9,10,11]. The extensive reliance on host proteins for viral replication is a common feature of RNA viruses and, in the case of HIV-1, additional clinical complications arise due to specific interactions with other viruses [12]. These include enhanced viral expression via the Tat protein or indirectly mediated by cytokines [13]. The modulation of this interaction network in a virus-specific manner while maintaining host-relevant interactions would be an ideal therapeutic scenario expected to avoid the emergence of resistant mutants because cellular factors essential for the virus have a much lower intrinsic mutation rate. Here, we report the development and application of a biosafety-level-1 (BSL-1) compatible, high-throughput screening system that targets both viral proteins and all cellular partners involved in HIV-1 un-coating, intracellular trafficking, reverse transcription, nuclear entry and genome integration.

## 2. Materials and Methods

### 2.1. Cultivation of HEK293T-mCat1 and PM1-mCat1 Cells

Human HEK293T and HEK293T-mCat1 (see below) cells were maintained in DMEM supplemented with 2 mM glutamine, 100 U/mL penicillin G, 100 mg/mL streptomycin and 10% fetal calf serum (FCS). Human PM1 and PM1-mCat1 (see below) cell lines were maintained in RPMI-1640 medium supplemented with 2 mM glutamine, 1 mM sodium pyruvate, 100 U/mL penicillin G, 100 mg/mL streptomycin and 10% FCS. Upon reaching 80% confluence in T-75 flasks, HEK293T-mCat1 cells were washed, trypsinized and resuspended in the appropriate medium before seeding into 384-well plates. PM1-mCat1, a suspension cell line, was directly resuspended in the appropriate medium before seeding into 384-well plates. All cell cultures were performed in dedicated incubators at 37 °C in a humidified atmosphere with 5% CO_2_.

### 2.2. Cloning of Self-Inactivating Lentiviral Vectors Expressing Luciferase or mCat1

Firefly-luciferase expressing vector LeGO-Luc2 [Addgene Plasmid #154006] was created by replacing IRES-eGFP of LeGO-iG2 [Addgene Plasmid #27341] using BamHI and PciI(blunted) with Luc2-cDNA (Promega Inc., Madison, WI, USA) using BamHI and NotI(blunted) [14]. The murine Cat1 serving as a receptor of ecotropic murine leukemia virus (MLV) [15] was cloned into a LeGO-iZeo2 vector [16] to generate LeGO -mCat1-iZeo2 [Addgene Plasmid #154007]. To do so, Slc7a1, the gene encoding the murine Cat1, was PCR-amplified from SF1-mCat1-i-Neo [17], cloned into LeGO-iZeo2 using EcoRI/NotI and verified by DNA sequencing. LeGO -mCat1-iZeo2 co-expresses mCat1 and the zeocin resistance gene via an internal ribosomal entry site (IRES) [16].

### 2.3. Production of Viral Particles

Pseudo-typed lentiviral particles were generated as previously described [14]. Viral supernatants were produced by transient transfection of HEK293T packaging cells. Detailed protocols on production and titration of lentiviral particles are available at www.LentiGO-Vectors.de. To emphasize that the safety-optimized lentiviral vectors used in this assay do not replicate within the target cells, we use the term “transduction” (receptor-mediated uptake) instead of “infection”. The lentiviral vectors are not “infectious”, compared with wild-type HIV.

### 2.4. Generation of HEK293T-mCat1 and PM1-mCat1 Cells

To render the human cell lines HEK293T and PM1 susceptible to lentiviral particles pseudo-typed with ecotropic MLV envelope (Env), both cell lines were transduced with LeGO-mCat1-iZeo2 encoding the murine Cat1 receptor and thereafter selected with zeocin. HEK293T-mCat1 cells were then grown as single cell clones, whereas PM1-mCat1 cells were used as polyclonal culture. Both new cell lines were tested for their transducibility with ecotropic lentiviral vectors expressing a fluorescent protein. Obtained transduction rates of up to 95% proved their suitability for the use in infection assays compliant with BSL-1 conditions.

### 2.5. Viral Screening Using Fluorescence-Based Vectors

Fluorescence analysis was carried out using an Opera confocal laser scanning microscope (LSM) (PerkinElmer, Waltham, MA, USA). To this end, 50 nL of each compound was transferred to individual wells using an Echo550 (Labcyte Inc., Sunnyvale, CA, USA), and HEK293T-mCat1 cells were seeded in black 384-well CellCarrier plates (PerkinElmer) at densities of 1200 cells per well in 25 µL medium. Then, 25 µL of diluted lentiviral supernatant was added before incubating the plates for 2 days. The applied multiplicity of infection (MOI) of seven, relative to the titration, was selected to ensure moderate transduction rates of about 60% under assay conditions, balancing between sufficiently labelled cells and mostly single vector integrations per cell to ensure a high sensitivity of the assay. After incubation, the cells were fixed and stained by replacing the medium with TBS-T (10 mM Tris-HCl (pH 7.4), 150 mM NaCl and 0.1% Tween-20) containing 3% formaldehyde and 1.5 µM Hoechst33342 for the nucleic acid staining. After 20 min, the fixation/staining solution was replaced with TBS-T, and imaging was carried out using the Opera confocal LSM at 20-fold magnification. Images were analyzed at the single-cell level using Columbus v2.3.1 (PerkinElmer). Single-cell data from each well were averaged and used for further analysis.

### 2.6. Viral Screening Using Luciferase-Based Vectors

Transfer of the compounds with the Echo550 and cell seeding (1200 per well) were performed as above, this time in white 384-well CellStar microplates (Greiner BioOne, Frieckenhausen, Germany). Again, 25 µL of lentiviral supernatant was added before incubating the plates for 3 days. Lentiviral vector titers were adjusted to obtain ca. 50% of the maximal signal, which resulted in a 10-fold window between signal and background. Higher signal intensities were avoided as they would need higher viral titers which would lead to viral toxicity mediated by multiple vector copies per cell. Luciferase activity was measured using SteadyLite plus (PerkinElmer), which was prepared following the manufacturer’s protocol. Briefly, 25 µL of the reagent were added per well, incubated in the dark for 10 min, and luminescence intensity was measured using an Envision multimode reader (PerkinElmer).

### 2.7. Flow-Cytometry Based Small-Molecule Inhibitor Analysis

Cells were prepared differently, depending on the assay. If flow-cytometry (FC) analysis was performed as a QC measure with cells from the 384-well assay plates, the cells were trypsinized and expanded in 24-well plates. In case FC analysis was performed independently, 50,000 cells per well were seeded together with the test compound into 24-well plates. Then, 12 µL of the lentiviral supernatant mediating transduction rates of about 50–60% was added before incubating the plates for 3 days at 37 °C and 5% CO_2_. FC analysis was carried out on trypsinized cells resuspended in the corresponding analysis buffer in FC tubes (BD Biosciences, San Jose, CA, USA). Before measurement, cells were resuspended again and 20,000 cells were analyzed per condition using either a FACS Canto II or an LSR Fortessa (both BD Bioscience). PMEG hydrate was obtained from Sigma Aldrich (Ordering number M2199).

### 2.8. Library Selection for High-Throughput Screening

For the selection of a screening library from the 200,640-compound in-house library, all compounds had to be standardized by removing salts and solvates, as well as aligning aromatization and tautomerization. The chemical structure of a set of 21 known HIV drugs was assembled, including eight NRTIs, six NNRTIs and five integrase inhibitors. The set was then normalized, and a similarity analysis with a similarity coefficient of ≥0.7 was performed. This yielded only three compounds in the 200,000-set that had similarity with the known HIV drugs, and further compound selection was therefore based on the diversity analysis. The 200,640-set was assigned to 3000 clusters using a ECFP fingerprint with 1024-bit fingerprints, and an analysis was performed to identify the number of clusters appearing on each plate of compounds in the 200,640-set [18]. Fifteen plates with potential protein–protein interactor compounds from the in-house library were chosen based on the maximum number of clusters represented per plate, resulting in 5280 compounds. An additional 59 plates were selected from the remaining compound subsets (about 170,000 compounds) based on the maximum number of represented clusters, yielding 20,768 compounds. Software used for the analysis and identification of frequent hitters included ChemAxon “Instant JChem” and various attendant packages, Dotmatics “Vortex” and Optibrium “StarDrop”.

### 2.9. High-Throughput Screening Using Luciferase-Based Vectors

Luciferase-based screening was conducted using the fully-automated cell::explorer system (PerkinElmer, Waltham, MA, USA). PM1-mCat1 cells were seeded using a MultiDrop (Thermo Fischer Scientific, Waltham, MA, USA) at a density of 5000 cells per well in a total volume of 20 µL in white 384-well CellStar microplates (Greiner BioOne, Frickenhausen, Germany). Plates were incubated at 37 °C in a tissue culture incubator for 12 h. Thereafter, 62.5 nL of compound was added to each well using an Echo550. The addition of 5 µL of lentiviral supernatant using a MultiDrop (ThermoFischer Scientific) was followed by incubating the plates for 3 days. Lentiviral vector titers were adjusted to obtain 80% of the maximal signal minimizing transduction-related toxic effect. The maximal final DMSO concentration during the primary screening and hit confirmation was 0.5%. Luciferase activity was measured using SteadyLite plus, which was prepared according to the manufacturer’s protocol. Briefly, 10 µL of the reagent was added per well using a MultiDrop, incubated in the dark for 10 min, and the signal intensity was measured using an Envision multimode reader. The data were analyzed using ActivityBase (IDBS Ltd., Guildford, UK) and normalized to DMSO-treated cells (0%) and cells treated with 100 nM Raltegravir (100%).

### 2.10. Cytotoxicity Determination Using HEK293T-mCat1 and PM1-mCat1 Cell Line

To determine the cytotoxic activity of the tested compounds, a setup similar to that used for the primary screening was applied. Cells were seeded using a MultiDrop (Thermo Fischer Scientific) at a density of 1200 HEK293T-mCat1 cells or 7500 PM1-mCat1 cells per well in a total volume of 25 µL in white 384-well CellStar microplates (Greiner BioOne) and were incubated at 37 °C in a tissue culture incubator for 12 h. We added 62.5 nL of compound to each well using an Echo550 (Labcyte Inc.) and incubated them for 3 days. Toxicity was measured using CellTiter-Glo (Promega Inc., Madison, WI, USA), which was prepared according to the manufacturer’s instructions. Briefly, 10 µL of the reagent was added per well using a MultiDrop, incubated in the dark for 10 min, and the signal intensity was measured using an Envision multimode reader.

## 3. Results

### 3.1. Fluorescence-Based Proof-of-Concept Screen

**Figure 1:**
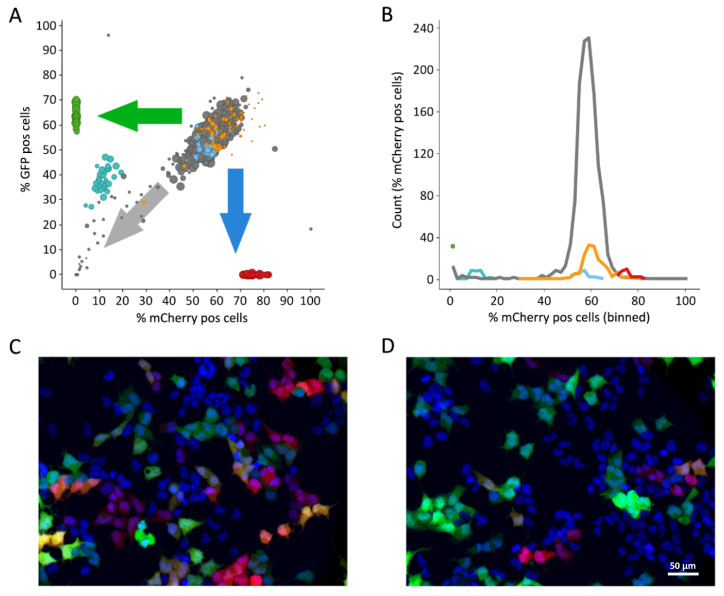
Fluorescence-based proof-of-concept screen. (**A**) The plot illustrates the proportion of red and green cells within all wells. Green data points reflect control wells transduced with the eGFP-expressing vector only (azidothymidine (AZT)-resistant mutant); red data points, with the mCherry-expressing vector only (wild-type strain). These two controls demonstrate the location of the data points, if a compound would completely inhibit just the mutant or the wild-type strain, respectively. The turquoise data points correspond to the co-transduced, AZT-treated wells and clearly demonstrate that the wild-type strain (mCherry) is more strongly inhibited by AZT than the AZT-resistant mutant (eGFP). Co-transduced, DMSO-treated wells served as negative controls and are shown in orange, co-transduced untreated wells in light blue. Co-transduced wells treated with the compounds to be analyzed are depicted in grey. Compounds inhibiting both strains are located in the direction of the grey arrow, with the most effective compounds being close to the origin of the plot, at 0% eGFP and 0% mCherry. Compounds inhibiting the wild-type strain only would be located in the direction of the green arrow, whereas compounds inhibiting the AZT-resistant mutant only would be located in the direction of the blue arrow. An intermediate inhibition characteristic is given with the AZT control (turquoise data points), mostly but not exclusively inhibiting the wild-type strain. Cell viability is illustrated by the size of the data points. (**B**) Frequency distribution of the fluorescence-based screening using the same color coding as in A. (**C**) Fluorescence microscopic image of co-transduced HEK293T-cells within a DMSO-treated control well. EGFP-positive cells (AZT-resistant mutant) are depicted in green, mCherry-positive cells (wild-type strain) in red, nuclei are stained in blue. Double-positive cells show a color gradient from orange to yellow based on expression levels. Scale bar as in D. (**D**) Fluorescence microscopic image of co-transduced HEK293T-cells within an AZT-treated well, less mCherry-positive cells are visible. Color coding as in C.

For proof of concept, we first applied a previously developed assay system based on the co-transduction of cells with two types of lentiviral particles, one depending on the wild-type HIV-1 reverse transcriptase (RT) and the other on an AZT-resistant RT mutant. They can be distinguished after transduction based on the expression of either the red fluorescent protein mCherry or the enhanced green fluorescent protein (eGFP), respectively [19]. Following co-transduction, high-content imaging was used to differentially identify compound activity with regard to the HIV-1 RT mutant status in combination with the viability parameters. The transduction efficiencies measured by either FC or microscopy were comparable, despite the reduced cell numbers analyzed in the image-based assays [19]. The co-transduction assay was performed at around a 60% transduction rate, whereas single-transduction controls were 10% higher on average (Figure 1A,B). We used 1 µM AZT as a control to demonstrate the reduction in the relative number of mCherry-positive cells, indicating the differential sensitivity of the two particle types (Figure 1C,D), as reported previously [19].

The screening was performed using the LOPAC library of 1280 drug-like compounds, each at a concentration of 1 µM. The average z’ of the four 384-well plates was 0.62, and the standard deviation of the transduction rates for the high controls was 7% on average, confirming the robustness of the assay system. Although most compounds did not affect the transduction efficiencies as evident from comparison of the DMSO-treated and compound-treated wells (Figure 1B), 35 compounds reduced the transduction rates of at least one of the two lentiviral particle types to less than 30%, and these were regarded as active. The majority of the identified active compounds also reduced the numbers of viable cells to ≤70% and were therefore classified as cytotoxic agents (Figure S1), but four compounds showed no signs of toxicity (Figure S1). Strikingly, three of these four compounds, namely PMEG, cytosine-1-β-d-arabinofuranoside (Ara-C) and AZT, are known virostatics, which underlines the suitability of the high-content screening approach (Table S1) [20,21]. The fourth compound, fenoldopam bromide, was previously described as a selective partial D1 receptor agonist and is used as an antihypertensive agent [22,23]. Interestingly, none of the eight other compounds described as having antiviral activity in the LOPAC library were active in the screening. This was due to their specific mode of action and therefore not a false negative result of the screening campaign (Table S2). The specificity of the assay was not compromised as none of the compounds should have been picked up. After demonstrating its general applicability, we next aimed at improving the assay in terms of throughput and biological relevance.

### 3.2. Luciferase-Based Screening Using HEK293T-mCat1 Cells

**Figure 2:**
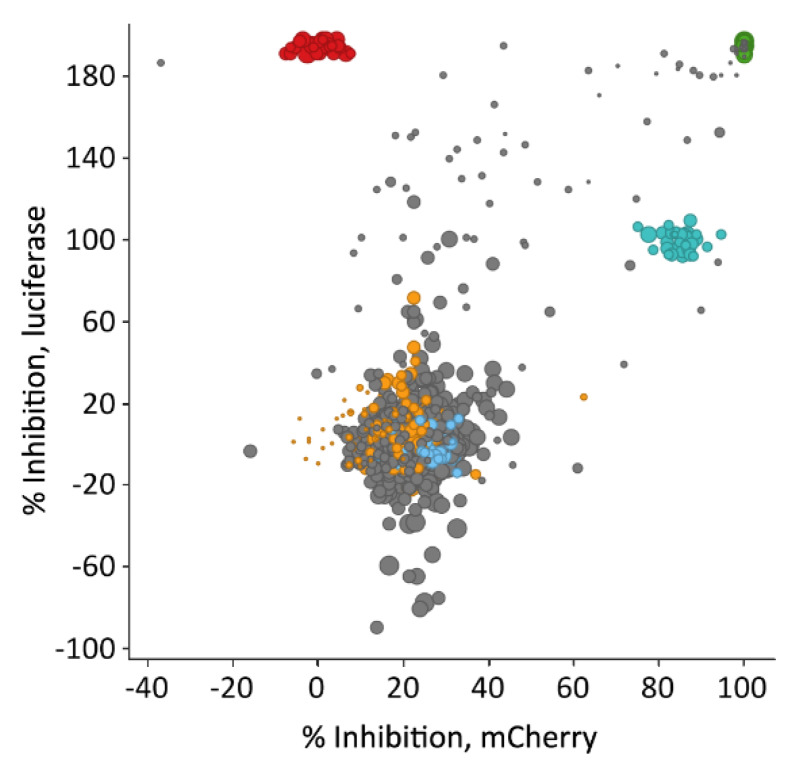
Luciferase-based proof-of-concept screen. Comparison of luciferase-based and mCherry-based wild-type reverse transcriptase data set. Data on the *y*-axis show luciferase inhibition. Data were normalized to AZT-treated wells, set to 100%, and DMSO-treated wells, set to 0% inhibition. Cells not expressing luciferase are at 200% inhibition, as AZT is reducing the signal by 50%. Color coding is as Figure 1: Transduced DMSO-treated wells in orange, transduced compound-treated wells, which were screened in both setups, in grey, transduced AZT-treated wells in turquoise and mCherry-only transduced cells in red, eGFP-positive cells shown in green. Viability is illustrated by the datapoint size.

To improve throughput and stability while simultaneously reducing the costs per well, we replaced the fluorescence readout by a luciferase-based system. After adjusting the number of viral particles needed for the optimal measurement, we optimized the assay to reduce the standard deviation from 7% for the fluorescence-based readout to less than 4%. This led to an improved stability of the system, resulting in an average z’ factor of 0.65 for the second LOPAC screen (i.e., with the same compounds as above). AZT, at a concentration of 1 µM, was used as a control and set to 100% inhibition in this screen. As this concentration did not completely block transduction, compounds with a higher activity achieved inhibition values greater than 100%. This screen revealed a number of additional active substances, many of them only active in the luciferase assay (Figure 2).

In fact, the new compounds exhibited a strong luciferase inhibition, but only marginally reduced the relative number of mCherry-positive cells in the fluorescence-based assay. Together, these findings suggested that the respective compounds probably interfered with the luciferase readout, e.g., by directly inhibiting the enzyme. Importantly, the three above-mentioned virostatic compounds PMEG, Ara-C and AZT were again identified in the luciferase assay, whereas fenoldopam bromide was not (Table S1). It might be speculated that fenoldopam inhibits the fluorescence proteins or their maturation as it also inhibits the different fluorescence proteins to the same extend.

In summary, the adaptation of the assay system to a luminescence-based readout increased the overall stability and enabled high-throughput screening. The loss of the possibility to assess compound activity against different virus mutants in one single well is counterbalanced by the much faster readout time; less than 2 min per plate compared with 1 h per plate in the fluorescence assay format.

### 3.3. Development of a Luciferase-Based Screening Assay Using PM1-mCat1 Cells

In the next step, we aimed at increasing the biological/pharmacological significance of our screening platform by adapting it to human CD4+ T cells, the natural target of HIV. To do so, we generated a PM1 cell line stably expressing the mCat1 receptor. PM1 was derived from HUT-78, a human cutaneous T-cell lymphoma line and is a commonly used model in HIV research [24]. We assumed that such a cell line would be a physiologically more relevant cellular model for HIV infection and might therefore facilitate the identification of not only compounds that interfere with viral proteins, but also modulators of cellular processes required for different steps of the viral life cycle (until integration).

**Figure 3:**
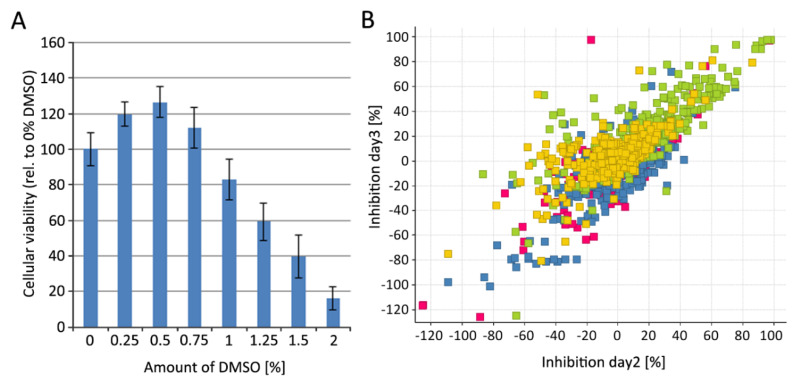
PM1-mCat1 cell-based assay development. (**A**) Viability of the PM1-mCat1 cell line in relation to DMSO concentration. (**B**) Marker library consisting of 1320 compounds was screened at 5 μM. PM1-mCat1 cells incubated for two days with compounds and viral particles are plotted against a 3-day incubation. Color coding is in accordance with plate numbers. First plate is shown in red, second in blue, third in green and the fourth plate is shown in yellow.

In the course of assay development, we optimized various parameters, including cell numbers, assay volume, amount of viral vector, reagent volume, variability and DMSO tolerance (Figure 3A, complete data not shown). The optimal timing and compound concentrations for high throughput screening (HTS) were determined by marker library screening at 10 µM and 5 µM, for 2 and 3 days (Figure 3B). The marker library consisted of four plates of a representative and diverse subset of the compounds to be screened in the primary screening. Overall, the impact of incubation time was limited, as indicated by the similar hit rates, i.e., about 12% at 10 µM and about 6% at 5 µM, when screened for 2 or 3 days, although a small subset of compounds showed higher activity, if incubated for 3 days. These compounds appear to be shifted upwards in Figure 3B.

At the end of the assay development, marker library screening was performed at two days and two concentrations. Now, the more potent Raltegravir was used as the control, resulting in minor differences in activity. Using this strategy, we were able to achieve an average 40-fold signal to the background window and a z’ value of 0.78. Raltegravir IC50s decreased from 2.1 to 7.9 nM during a 6-month period, most likely due to minor stability issues as cells were kept at low passage and replaced with cryo-preserved ones if needed.

### 3.4. Step-Wise Establishment of a BSL-1 Compatible HTS Assay

Here, we aimed at establishing high-throughput screening for the identification of small molecules with anti-HIV-1 activity. Toward this aim, we first developed a fluorescence-based assay system suitable for biosafety level-1 (BSL-1). This was achieved by pseudo-typing HIV-1-derived lentiviral vectors with the envelope protein of ecotropic mouse leukemia virus (MLV), which is unable to bind to human cells [25]. These “mouse only” viral particles could still be used in human cells after the murine Cat1 symporter, which serves as the receptor for ecotropic MLV, was stably expressed in HEK293T cells. The resulting, very robust assay facilitated the assessment of three relevant parameters in parallel: activity against “wild-type” viral particles, activity against particles with mutant RT and cytotoxic activity.

**Figure 4:**
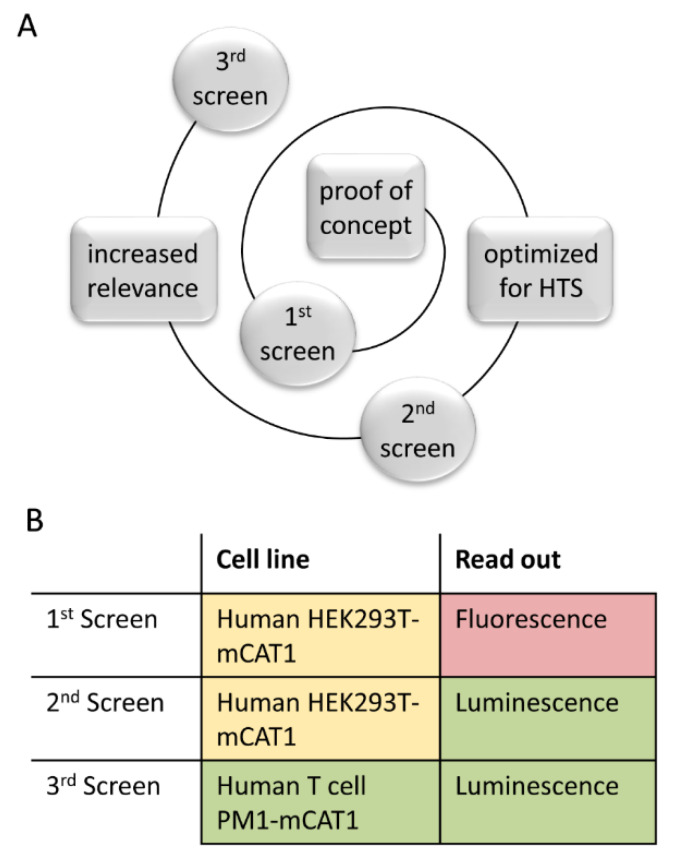
Overview of the assay methodology development. (**A**) Representation of the assay development based on incremental improvement. (**B**) Comparison of the screening methodology in terms of relevance, based on a traffic light system.

As fluorescence-based assays intrinsically exhibit a lower sensitivity and a lower signal-to-noise ratio, but also require a quite time-consuming readout, we switched to a luciferase-based readout in a second round of screening (Figure 4A) [26,27]. To do so, we made use of the modularity of the lentiviral LeGO vector system which allows easy transgene switching. The second generation of the assay was also developed using the human HEK293T-mCat1 cell line, but now a luciferase gene provided by the lentiviral particles was used as the reporter. The PM1-mCat1 cell line used in the third generation of the assay (Figure 4B) is a derivative of the human T cell line PM1, a standard cell line broadly used in research of HIV biology. Closely mimicking the normal infection route is especially important for the envisioned approach, as we aimed to target cellular factors present in the assay cells at physiological concentrations.

In summary, we developed and step-wise improved a BSL-1 compatible screening approach resulting in a physiologically relevant assay, highly compatible with high-throughput screening (Figure 4). This enabled us to go on and perform a large-scale screening using industrial standards and a cellular model, which will deliver meaningful data.

### 3.5. Library Selection and Screening Using the PM1-mCat1-Based HTS Format

The screening library was selected from the 200,640-compound in-house library using a dual approach. The final selection was limited to about 25,000 compounds and oriented towards diversity but also towards known HIV drugs to further validate the assay system. The structure of a set of 21 known HIV drugs, consisting of NRTIs, NNRTIs and integrase inhibitors, was assembled, and a similarity search revealed three analogs in the library, which were included in the screening library. The complete library of 200,640 compounds was also divided into 3000 clusters based on chemical diversity. Compound plates were chosen according to the maximum number of clusters per plate, resulting in a screening collection of 26,048 compounds from 2723 clusters. This highly diverse set, consisting of 81 plates, was screened at a 5-µM compound concentration on two consecutive days with an average z’ value above 0.8.

**Figure 5:**
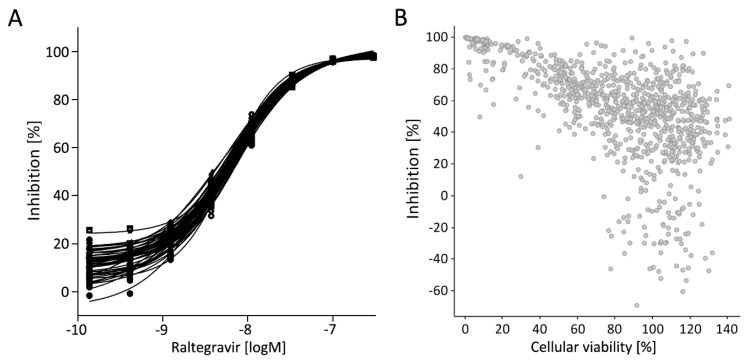
PM1-mCat1 cell-based assay development. (**A**) Raltegravir dose–response curves at one representative primary screening day as an indicator of the reproducibility of the assay. (**B**) Comparison of primary activity as % luciferase inhibition and toxicity, measured by ATP-determination, in PM1-mCat1 cell line. Data points in the top right area of the plot represent compounds showing high inhibition and low toxicity. Data points in the top left area represent compounds showing high inhibition and high toxicity.

The Raltegravir pharmacology control of the first screening day, consisting of 50 plates, confirmed the high degree of reproducibility of the assay system (Figure 5A). DMSO-treated cells were set to 0% activity, whereas cells treated with 100 nM Raltegravir were set to 100% activity and compounds with an activity below −45% (activators) or above 66% (inhibitors) were classified as hits. The primary hit rate was at 3.3%, and the active compounds were validated by hit confirmation. To rule out an indirect impact on virus integration, toxicity against the screening cell line was analyzed (Figure 5B). A trend between both parameters was observed and active but non-toxic compounds, with a viability of >80% and an activity of <−45% or >60%, appear in the lower or upper right quadrant of the diagram (Figure 5B). The active compounds were further filtered by selecting those with very high consistency, having a standard deviation within the confirmational screening of less than 10%, resulting in 10 activators and 90 inhibitors, which were further filtered by applying an algorithm against classical assay interfering compounds. Overall, a series of 9 activators and 84 inhibitors were selected. This compound set was enriched by 279 similar structures taken from the whole library, three similar compounds per hit, and subjected to hit profiling.

### 3.6. Hit Profiling

For the initial validation, we carried out a parallel profiling of the PMEG hydrate using FC as an orthogonal readout. The FC-based analysis was able to reproduce the trend of the fluorescence-based high-content screening data (see above) at the screening concentration of 1 µM (Figure S2). On the other hand, the microscopy approach resulted in residual transduction rates of 5% and 30% for the eGFP and mCherry encoding lentiviral particles, respectively, and flow cytometry reported 30% and 60% residual transduction efficiencies. This discrepancy may reflect the different methodologies and plate formats or the newly ordered batch of the compound, as it was needed in a larger quantity for the FC assay. As an additional comparison, the 31 most active compounds from the HTS campaign were also selected for profiling using conventional FC analysis with VSV-G pseudo-typed viral particles to exclude false-positive inhibitors targeting the pharmacologically irrelevant Eco/mCat1 receptor interaction or the luciferase detection system. Despite a similar shift in activity as observed for the PMEG hydrate, the top five compounds showed up again as the five most potent inhibitors in almost the same rank order. It is worth mentioning that the orthogonal assay used to validate the results also confirmed that not the receptor interaction, developed for the BSL-1 approach, was targeted by the molecules, but rather a cellular or viral factor, thereby emphasizing the great potential of our approach.

**Figure 6:**
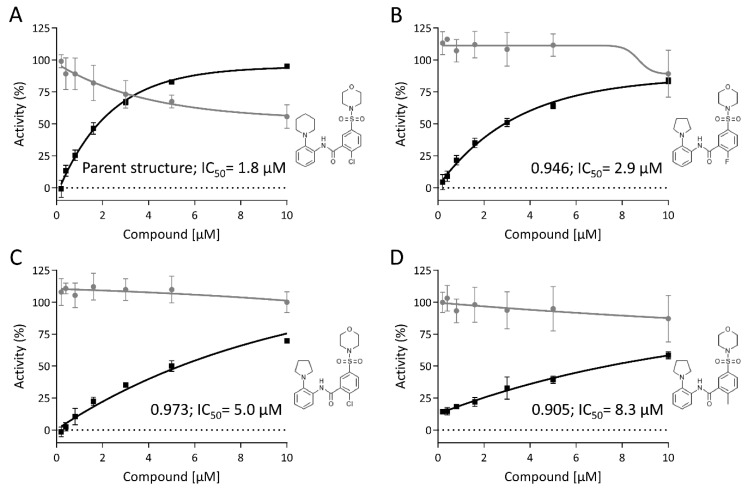
Example of four similar inhibitors showing structure-specific differences in activity (**A**–**D**). Cellular viability relative to the DMSO-treated control is shown in grey. Inhibition of virus integration is shown in black. The compound structures are given next to the inhibition curves. The numbers next to the curves indicate the similarity based on the tanimoto coefficient and the IC_50_ in the virus integration assay.

The inhibitor dataset, which was analyzed further in dose–response studies, consisted of 84 small molecules. This inhibitor collection was enriched with three analogues per compound, selected via a similarity search from the in-house compound collection. From these 84 clusters, 33 contained one active compound only, but 26 clusters contained two, eight clusters three and in four clusters, all four compounds were active. Interestingly, this selection was enriched for certain chemotypes which could be traced back to the initial hit list. A substituted tetrazole, which was found in 0.5% of the compounds from the initial screening set, was enriched to 8% of the compounds in the hit list. Another example is the thieno[2,3-D]pyrimidine substructure, which was enriched from initially 0.5% to 7%. These examples show that the high-throughput screening approach revealed inhibitors that were active in an orthogonal assay format and also consisted of different chemotypes, therefore potentially modulating different targets. Figure 6 depicts one of the clusters with four active compounds. The parent structure of the cluster 6 was toxic beyond 2 µM, as the viability did drop below 80%, but the other compounds of the cluster were non-toxic. The activity of selected hit compounds including cluster 6 (Figure 6) is given in Table S3.

## 4. Discussion

We developed a novel assay to screen for HIV-inhibiting compounds. Since our assay is based on living cells, it allows to identify inhibitors not only directed against HIV, but also directed against cellular targets. To allow a broad application, the screening cascade presented here (Figure 4) was adapted to BSL-1 conditions. Initially, we used the HEK293T-mCat1 cell line, stably expressing the mCat1 receptor, as a screening host for high-content screening. The presence of the membrane receptor mCat1 on the cell surface allows the entry of lentiviral particles pseudo-typed with the ecotropic MLV env protein. Thus, our assay combines the species-dependent criteria necessary to comply with biosafety level 1 and the functionality and speed of lentiviral systems [25]. The safety-optimized HIV-1 derived, lentiviral vectors still use the same mechanisms and viral proteins for important stages of the HIV life cycle, including membrane fusion, reverse transcription and integration of the provirus into the host cell’s DNA. After integration, the expression of the marker genes (here, eGFP, mCherry or luciferase) allows for an unequivocal identification and quantification of successful integration events, which corresponds to infected cells, without the need to handle the infectious HIV wild-type virus. Thus, the utilization of the marker genes enables straightforward analysis methods at high speeds and low costs. The system using pseudo-typed lentiviral particles could also easily be adapted to screen other relevant cell lines or other retroviruses.

The first generation of the screening cascade based on the high-content image analysis provided multiplexing capabilities, a clear benefit compared with similar fluorescence-based methodologies, normally analyzed using multimode plate readers [28]. The assay performed well, as seen by the reported IC50 for the PMEG hydrate of 200 nM, which is generally in line with the 5% residual activity identified at 1 µM in the high-content screening (Table S1) [29]. Notably, the FC-based analysis of the PMEG hydrate showed a reduced activity, as the IC50 of the AZT-resistant mutant was 1.2 µM and 560 nM in the case of the wild-type virus (Figure S2). The IC50 of Raltegravir determined during the assay development of the third assay generation was 2.1 ± 0.9 nM, which was in excellent accordance with the cellular IC50s between 0.5 and 1.5 nM and the biochemical IC50s between 2 and 5 nM reported in the literature [30,31].

The observed overall screening statistics with average z’ values above 0.8 are well suited for HTS. This is in the same range with other HTS-compatible, HIV-1 inhibitor screenings using reporter virus assays or reporter cell assays [32,33,34]. A complete overview of the hit and confirmation rates of the different screens is given in Table S4. Reporter cell assays that have been used are based on primary cells, established T cells or fibroblasts, engineered to express a reporter gene upon viral replication [3]. Those systems tend to have a number of drawbacks, namely the safety requirements due to the use of replication-competent HIV, the artificiality of the engineered cell line and the reduced sensitivity of the assay compared with replication-deficient viral particles. Engineered cell lines, like HEK293 or HeLa cells, are frequently used as reporter cell lines as their adherent cell growth simplifies fluorescence-based readouts [28]. However, they do not represent a cell type naturally infected by HIV-1 and therefore respond non-physiologically, in contrast to PM1 cells [33]. Moreover, the lack of tight promoter control resulted in a high background signal and thus reduced the sensitivity of the reporter cell assay [28,35]. Reporter virus assays have been suggested as viable alternatives. As they are frequently single infection assays and do not facilitate virus spread between cells, the incorporated reporter gene needs to warrant high sensitivity [34,36]. Using the luciferase reporter gene, we confirmed that this is the system of choice. Another type of high-throughput assays aims at identifying novel drugs to eliminate latently HIV-infected cells, acting as long-term virus reservoirs [37].

An additional advantage of virus reporter assays is the possibility to identify compounds enabling a more efficient viral entry. Even though this would obviously not facilitate the finding of new HIV-1 therapeutics directly, it might help to discover the relevant pathways and interaction partners. Furthermore, in the field of gene therapy, efficient transduction is a sine qua non. This becomes particularly relevant when working with large gene inserts that are at the limit of the packaging capacity, resulting in low virus titers [38]. We therefore also confirmed the efficiency of 10 activators identified during screening, possibly leading to viral transduction enhancers.

In summary, we have developed an efficient screening cascade to identify early stage HIV-1 inhibitors in a high-throughput, cost-effective and BSL-1 compatible environment, and we identified a number of promising compounds which are currently being developed further. The target spectrum of this assay cascade is not limited to viral proteins, widely studied for many years, but potentially also includes cellular factors which might represent very interesting targets. Compared with viral proteins, cellular proteins do not face selection pressure and are mostly invariant among different patient groups. Thereby drugs against cellular proteins are likely to be effective against all viral subclasses and less susceptible to resistance development. The naturally occurring deletion variant of CCR5, an HIV co-receptor, has provided proof of principle that the modulation of cellular factors can effectively block HIV infection without impairing human physiology [39]. Another example is the class of entry inhibitors, e.g., TNX-355 or maraviroc, which bind or mimic cellular factors and thereby inhibit viral entry [40,41]. Moreover, genome-wide association studies have shown that variants of the HLA-gene are also significantly linked to low viral titers and extended periods between infection and AIDS outbreak [42].

Finally, small-molecule modulators have already proven the feasibility of modulating the specific protein function in viral infections. For instance, inhibiting the interaction of protein phosphatase-1 with the HIV-Tat protein, but not the interaction with important cellular targets (NIPP1 and PNUTS), HIV-1 replication in MT4 cells can be blocked [43]. A 700,000-compound screen identified an inhibitor of viral integration by specifically restricting the interaction between the cellular LEDGF/p75 and the viral integrase [44,45]. Another example is amphotericin B methyl ester, a HIV-1 replication inhibitor with a previously unidentified mode of action. It was recently shown to interfere with the HIV-Vpu activity, which normally reduces the ability of tetherin to inhibit HIV-1 budding [46]. These examples demonstrate the feasibility of the host-based approach.

The screening cascade presented here will thus clearly trigger a number of projects that advance our understanding of cellular factors interacting with HIV-1 that can be targeted in novel therapeutic approaches.

## Supplementary Materials

The following are available online at https://www.mdpi.com/1999-4915/12/5/580/s1, Figure S1: Fluorescence-based proof-of-concept screen, Figure S2: Activity of PMEG hydrate analyzed in FC-based assay. Table S1: Hits of the fluorescence-based proof of concept screen compared to the luciferase-based data, Table S2: Compounds with antiviral mode of action in the LOPAC library which appeared to be not active in the fluorescence-based proof of concept screen, Table S3: Hit compounds with an IC50 beyond 1 µM in viral integration assay and their structural information.
