# Supplementary material for: A High-Throughput HIV-1 Drug Screening Platform, Based on Lentiviral Vectors and Compatible with Biosafety Level-1
Source: Viruses. 2020 May 25;12(5):580. doi: 10.3390/v12050580 (PMC7290285; doi:10.3390/v12050580)
Supplement: Supplementary file 1 [file viruses-12-00580-s001.pdf]

viruses

MDPI

Supplementary Material

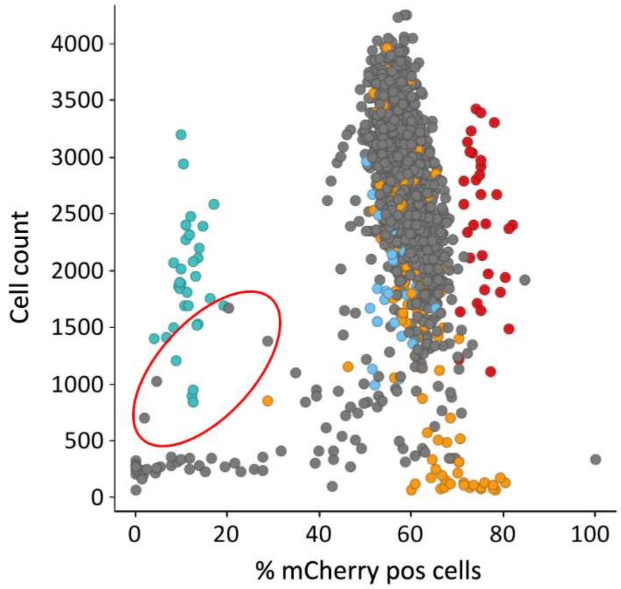

Figure S1: Fluorescence-based proof-of-concept screen. Relative number of transduced cells, data shown as average per well. Co-transduced compound-treated wells shown in grey and co-transduced AZT-treated wells in turquoise. Cells transduced using mCherry only are shown in red, co-transduced DMSO-treated wells in orange and co-transduced untreated wells in light blue. Compound-treated wells encircled in red represent the four most promising hits of the screening.

Table S1: Hits of the fluorescence-based proof of concept screen compared to the luciferase-based data. The negative inhibition value of Fenoldopam bromide is due to an increase of the luciferase signal in the screen. The inhibition value above 100% in explained by the AZT normalization of the assay, as the internal control is not abolishing the luciferase signal.

Compound	Relative inhibition [%] of			Viability [%]*
	eGFP (mutant RT)	mCherry (wild-type RT)	luciferase (wild-type RT)	
Cytosine-1-beta-D-arabino-furanoside hydrochloride	95	98	211	38
PMEG hydrate	70	94	156	54
Fenoldopam bromide	65	61	-40	73
3′-Azido-3′-deoxythymidine	38	73	85	89

Table S2: Compounds with antiviral mode of action in the LOPAC library which appeared to be not active in the fluorescence-based proof of concept screen. The table lists all the compounds from the LOPAC library with an antiviral mode of action, which were not identified as hits, as well as the data of the screen and an explanation if the compound has to be counted as false negative, e.g. if the assay should have identified this compound as active.

Compound	Descrition within LOPAC	Rel. inhibi-tion [%] of eGFP	Rel. inhibi-tion [%] of	Via-bilit y [%]	Explanation in the context of the assay. False negative

		**(mutant RT)**	**mCherry (wild-type RT)**		
Amanta-dine hydro-chloride	Dopamine releaser used in the treatment of Parkinsonism and drug-related extrapyramidal reactions; antiviral (Influenza A)	-29	6	67	The compound class was also tested against HIV and found to work via a membranotrophic mode of action. Although it can be active against HIV in general, it can be imagined that the murine receptor used during screening is not responsive to this type of inhibition. The compound was identified correctly as not active.
Acyclovir	Antiviral agent	-33	41	88	Acycloguanosine (Aciclovir) is a prodrug, requiring a viral thymidine kinase not present in HIV to be activated. The compound was identified correctly as not active.
2',3'-Dideoxy-cytidine	Nucleoside analog; reverse transcriptase inhibitor; antiviral	99	100	7	The compound ddC (Zalcitabin) is a nucleosidic reverse transcriptase inhibitor. In this assay the compound was identified as toxic and therefore not followed up. The compound was identified correctly as active and toxic and was therefore not pursued further.
3-Deaza-adenosine	Antiviral	-34	-25	85	The nucleoside analog 3-Deazaadenosine inhibits andenosylhomocysteine hydrolase and thereby the methylation of membrane phospholipids. In the case of HIV it acts on endocytosis and viral entry and not on reverse transcriptase activity [1]. It can be imagined that the murine receptor used during screening is not responsive to this type of inhibition. The compound was identified correctly as not active.
Nelfinavir mesylate hydrate	Nelfinavir is a HIV protease inhibitor; antiretroviral; and anti-tumor agent	49	90	26	Nelfinavir is a HIV protease inhibitor, which is not active in the screen as HIV protease activity is not part of the cascade the screen can analyse. The assay utilizes mature viral particles, not relying on protease activity. The compound was identified correctly as not active.
Famciclovir	Famciclovir is an antiviral	100	100	7	Famciclovir is a prodrug, requiring a viral thymidine kinase not

					present in HIV to be activated. The compound was identified correctly as not active.
Ribavirin	Antiviral agent; its metabolite, ribavirin 5'-phosphate, is an inhibitor of inosine monophosphate (IMP) dehydrogenase	-73	-21	81	The compound (Ribavirin) has a complicated mode of action, but is not effective against RNA viruses in single therapy [2,3]. The compound was identified correctly as not active.

Table S3: Activity in the viral integration assay and structural information of selected hit compounds.

Name	Cluster	IC_{50} (in μM)	Smile
T5407044	1	4.11	COC1=CC=C(C=C1)S(=O)(=O)N(CC1=CC(=CC=C1)C(F)(F)F)C1=C(OC)C=CC=C1
T5788505	2	1.99	CC(NC(=O)CSC1=C(C#N)C(C)=C(C)C(C)=N1)C1=CC=CS1
T5989363	2	7.91	CC(C)CCNC(=O)CSC1=C(C#N)C(C)=C(C)C(C)=N1
T5615894	3	2.93	CC1=CC=CN2C(=O)C=C(CN3CC(OC4=C3C=CC=C4)C(=O)N3CCCCC3)N=C12
T6329376	3	4.83	CC1=C(CN2CC(OC3=C2C=CC=C3)C(=O)N2CCCCC2)N=C(O1)C1=CC=CC=C1
T5938606	4	5.91	CCC1=CC=C(C=C1)C1=CSC(CN2C(CN3CCOCC3)=NC3=C(C=CC=C3)C2=O)=N1
T5938605	4	8.71	FC1=CC=C(C=C1)C1=CSC(CN2C(CN3CCOCC3)=NC3=C(C=CC=C3)C2=O)=N1
T6496714	5	2.44	COC1=CC(OC)=C(C=C1)C1CCCN1C(=O)C1CN(C(=O)C1)C1=C(F)C=CC=C1
T6517641	5	6.83	COC1=CC(C2CCCN2C(=O)C2CN(C(=O)C2)C2=CC=CC=C2)=C(OC)C=C1
T6098586	6	1.78	ClC1=C(C=C(C=C1)S(=O)(=O)N1CCOCC1)C(=O)NC1=C(C=CC=C1)N1CCCCC1
T6382120	6	2.86	FC1=C(C=C(C=C1)S(=O)(=O)N1CCOCC1)C(=O)NC1=C(C=CC=C1)N1CCCC1
T6137813	6	5.04	ClC1=C(C=C(C=C1)S(=O)(=O)N1CCOCC1)C(=O)NC1=C(C=CC=C1)N1CCCC1
T5530682	6	8.26	CC1=C(C=C(C=C1)S(=O)(=O)N1CCOCC1)C(=O)NC1=C(C=CC=C1)N1CCCC1
T6328287	7	3.89	COC1=C(C=CC=C1)N1CCN(CC1)C(=O)C1=C(O)C=CC(Cl)=C1
T5821319	8	1.58	O=C(NC1=CC2=C(OCO2)C=C1)C1=CC(=CC=C1)N1CCCC1=O
T5609721	8	4.12	O=C(NC1=CC2=C(OCCCO2)C=C1)C1=CC(=CC=C1)N1CCCC1=O
T5351795	9	4.64	CC(C)CCN1C=NC2=C1C(=O)N(CC(=O)NC1CC1)C(=O)N2CC1=CC=CC=C1

T5488769	9	9.06	CC(C)CCN1C=NC2=C1C(=O)N(CC(=O)NC(C)C)C(=O)N2CC1=CC=CC=C1
T5945790	10	6.78	O=C(COC1=C(C=CC=C1)C1=CC=CC=C1)NCC1=C(CN2C=CC=N2)C=CC=C1
T5339957	10	6.88	O=C(COC1=C(C=CC=C1)C1=CC=CC=C1)NCC1CCCCC1
T5781151	10	6.94	O=C(COC1=C(C=CC=C1)C1=CC=CC=C1)NCC1=C(CN2CCOCC2)C=CC=C1
T6213020	12	2.79	COC1=CC=C(NS(=O)(=O)C2=CC(=CC=C2)C(=O)NCC(N2CCCCC2)C2=CC=C(OC)C=C2)C=C1
T5643556	13	6.19	FC1=CC=C(C=C1)C1=CSC2=C1C(SC1=NC=C(C=C1)S(=O)(=O)N1CCOCC1)=NC=N2
T5413794	13	6.91	FC1=CC=C(C=C1)C1=CSC2=C1C(OC1=CC=C(C=C1)S(=O)(=O)N1CCOCC1)=NC=N2
T5650116	14	2.79	COC(=O)C1=CC=C(C=C1)C(=O)NC1=NC=C(C)S1
T6026407	15	2.73	C(N1CCOCC1)C1=NC2=C(C(=CS2)C2=CC=CS2)C(NN2CCCCC2)=N1
T5605915	15	4.67	C(NC1=C2C(SC=C2C2=CC=CS2)=NC(CN2CCOCC2)=N1)C1=CC=CC=C1
T6061512	15	6.48	C(NC1=C2C(SC=C2C2=CC=CS2)=NC(CN2CCOCC2)=N1)C1CCCO1
T6312425	15	>10	C(CC1=NC=CC=C1)NC1=C2C(SC=C2C2=CC=CS2)=NC(CN2CCOCC2)=N1
T6357587	16	8.3	O=C(CC1=CSC(=N1)C1=NC=CC=C1)N=C1SC=CN1CC1=CC=CC=C1
T5344625	17	1.72	O=C(CSC1=NC2=C(C=CC=C2)C(=O)N1CC1=CC=CO1)N1CCN(CC1)C1=CC=CC=C1
T6302141	18	2.24	CN(C1CCCCCC1)S(=O)(=O)C1=CC(=CC=C1)C#N
T5339669	18	5.2	CN(C1CCCCC1)S(=O)(=O)C1=CC(=CC=C1)C#N
T6468172	19	1.04	BrC1=C(C=CC=C1)C(=O)NC1=C(C=CC=C1)N1C=CC=N1
T6043692	19	4.11	OC1=C(NC(=O)C2=C(Br)C=CC=C2)C=CC=C1
T5952497	20	4.27	O=S(=O)(N1CCC(CCC2=CC=CC=C2)CC1)C1=C(C=CC=C1)C#N
T5432718	20	6.33	O=S(=O)(N1CCN(CC2=CC=C(C=C2)C2=CC=CC=C2)CC1)C1=C(C=CC=C1)C#N
T5823772	22	8.88	CC1=CC=CC(C)=C1NC(=O)CN1CCCC1C1=NC2=C(S1)C=CC=C2
T5989502	24	1.94	CC(=O)C1=C(NC(=O)CNC2=C(C=CC=C2)C2=CC=CC=C2)C=CC=C1
T5947021	24	5.61	CC1=C(NC(=O)CNC2=C(C=CC=C2)C2=CC=CC=C2)C=CC=C1
T5943324	24	7.79	ClC1=C(NC(=O)CNC2=C(C=CC=C2)C2=CC=CC=C2)C=CC=C1
T6366633	30	1.67	FC1=CC=C(C=C1)C1=CSC2=C1C(SCC1=CC=C(O1)S(=O)(=O)N1CCCC1)=NC=N2
T6471356	31	2.37	C=CCNC(=O)CN1CCN(CC1)C1=NC2=C(S1)C=CC=C2
T6203208	32	4.37	CC(C)C1=CC=C(C=C1)C1NC(=S)NC(C)=C1C(=O)OCC=C
T6312190	33	2.02	CC1=C(Cl)C(=C(C)C(Cl)=C1)S(=O)(=O)NCCCN1CCCC1=O
T5776163	37	4.7	CCOC1=C(C=CC=N1)C(=O)OCC1=NC2=C(C(C)=C(C)S2)C(N)=N1
T5983422	38	2.28	O=C1NC(CSC2=NN=C(O2)C2CC2)=NC2=C1C(=CS2)C1=CC=CO1

T5435331	39	3.14	COC1=CC=C(C=C1)N(CC1=CC=CC=C1)S(=O)(=O)C1=CC(=CC=C1)C(=O)NCC1=CC=NC=C1
T5869032	40	1.1	CCOC1=CC=C(C=C1)C(=O)NC1=C(F)C=CC(C)=C1
T5506295	40	1.77	CC1=CC(NC(=O)C2=CC=C(OC(F)F)C=C2)=C(F)C=C1
T5950416	42	2.08	CS(=O)(=O)C1=CC=C(NC(=O)CNC2=C(C=CC=C2)C2=CC=CC=C2)C=C1
T5941648	42	7.85	CC(=O)C1=CC=C(NC(=O)CNC2=C(C=CC=C2)C2=CC=CC=C2)C=C1
T6332191	43	1.42	CC1=CC2=C(N=C1)C(=CC=C2)S(=O)(=O)N1CCN(CC2=CC3=C(OCCO3)C=C2)CC1
T6053460	47	6.46	COC1=CC=C(CC(=O)NC2=CC=C(OC)C=C2)C=C1
T6007055	49	1.35	COC(=O)C1=C(NS(=O)(=O)C2=C(Br)C=C(F)C=C2F)C=CC=C1
T5989159	51	1.67	CCC(C)C1=CC=C(C=C1)S(=O)(=O)NC1=C(C)C=C(F)C=C1
T5805638	52	1.45	CC1=C(C=CC=C1)N1N=NN=C1SCC1=NN=C(O1)C1=CC=CS1
T0516-3343	53	6.07	CCNC(=O)COC(=O)C1=C2C=CC=CC2=NC(=C1C)C1=CC(Cl)=CC=C1
T5633521	56	1.05	CCNC1=NN=C(CS1)C1=C(C)N(C2CC2)C(C)=C1
T5932815	60	2	CC1CCN(CC1)C1=C2C(SC=C2C2=CC=CS2)=NC(CN2CCOCC2)=N1
T5587925	60	3.97	C(N1CCOCC1)C1=NC2=C(C(=CS2)C2=CC=CS2)C(=N1)N1CCCC1
T5952126	61	1.53	CC(NC1=C2C3=C(CCC3)SC2=NC(CN2CCOCC2)=N1)C1=CC(Cl)=CC=C1
T5964141	65	1.26	O=C(CSC1=NN=C(O1)C1CC1)NC(C1=CC=CS1)C1=CC=CC=C1
T6068357	66	1.49	CCN1C(=O)C2=C(C=CS2)N=C1SCC(=O)NC1=C(C=CC=C1)C#N
T6671011	68	4	C(NC1=C(C=CC=C1)N1C=CC=N1)C1=NC(=NO1)C1=CC=CO1
T5595439	71	2.98	CC1=CC(C)=C(C=C1)N1N=NN=C1SC1=NN=NN1C1=CC=CC=C1
T5258076	76	4.76	FC(F)(F)C1=C(C=CC=C1)N1C(SCC(=O)NCC2=CC=CO2)=NN=C1C1=CC(Cl)=CC=C1
T5363639	79	2	ClC1=CC(NC2=C3C(SC=C3C3=CC=CC=C3)=NC(CN3CCOCC3)=N2)=CC=C1
T5948460	79	2.33	C(CC1CCCO1)NC1=C2C(SC=C2C2=CC=CC=C2)=NC(CN2CCOCC2)=N1

Table S4: Hit and confirmation rates of the different screening campaigns. The table lists the screening statistics of the different screening campaigns performed during different phase of assay optimization. A low confirmation rate does not imply a poor screening performance, but reflect the additional filter applied.

Assay	Compounds screened	Criteria for hit identification	Actives (hit rate in %)	Criteria for hit progression	Confirmed actives (hit rate in %)
Fluorescence-based proof of concept screening	1,280	Reduction of at least one transduction rate to less than 30%	35 (2.7%)	Confirmed activity and more than 70% viable cells after treatment	4 (11.4%)
Luciferase-based proof of concept screen	1,280	Reduction of the transduction	68 (5.3%)	Confirmed activity and more than	41 (60.3%)

		rate to less than 30%		70% viable cells after treatment	
Luciferase based high throughput screening	26,048	Reduction of the signal by at least 60% compared to the controls	930 (3.6%)	Reduction of the signal by at least 66%, standard deviation below 10%, viability above 80%	456 (49.0%)

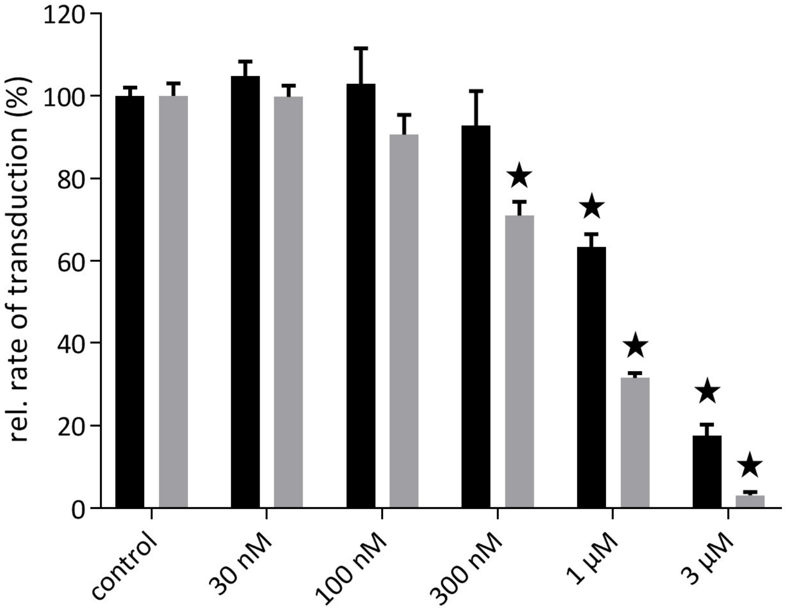

Figure S2: Activity of PMEG hydrate analyzed in FC-based assay. Dose-response analysis of PMEG hydrate using flow cytometry. Data are represented relative to DMSO-treated control. PMEG hydrate concentrations resulting in a significant deviation from the control are marked using an asterisk (t-test, $p<0.001$). Higher concentrations were excluded due to compound toxicity. Cells transduced using eGFP-encoding vectors (mutant reverse transcriptase) are shown in black, mCherry-only transduced cells (wild-type reverse transcriptase) in grey.

References

1. Flexner, C.W.; Hildreth, J.E.; Kuncl, R.W.; Drachman, D.B. 3-Deaza-adenosine and inhibition of HIV. Lancet 1992, 339, 438, doi:10.1016/0140-6736(92)90133-n.
2. Carrillo-Bustamante, P.; Nguyen, T.H.T.; Oestereich, L.; Günther, S.; Guedj, J.; Graw, F. Determining Ribavirin's mechanism of action against Lassa virus infection. Sci. Rep. 2017, 7, 11693, doi:10.1038/s41598-017-10198-0.
3. Lau, J.Y.N.; Tam, R.C.; Liang, T.J.; Hong, Z. Mechanism of action of ribavirin in the combination treatment of chronic HCV infection. Hepatology 2002, 35, 1002–1009, doi:10.1053/jhep.2002.32672.

© 2020 by the authors. Submitted for possible open access publication under the terms and conditions of the Creative Commons Attribution (CC BY) license (http://creativecommons.org/licenses/by/4.0/).
